# Dislocation of the Shoulder Joint – Radiographic Analysis of Osseous Abnormalities

**DOI:** 10.5334/jbr-btr.1210

**Published:** 2016-11-19

**Authors:** Bruno Vande Berg, Patrick Omoumi

**Affiliations:** 1 Cliniques Universitaires Saint Luc, BE; 2 University Hospital of Lausanne, CH

**Keywords:** Shoulder, Instability, Impact fracture, Bone, Dislocation, Radiography

## Abstract

Radiography remains pivotal to the workup of instability lesions of the shoulder, both in the acute as well as the chronic settings. The goal of radiography is to detect osseous abnormalities and locate them in order to determine the direction of instability. In antero-inferior instability, Hill-Sachs lesions are often visible at radiography and should not be confused with various differential diagnoses, which are usually more laterally located. Bankart lesions are more difficult to detect on conventional radiography, but there are less false positives than for Hill-Sachs lesions. The Garth view represents an excellent radiographic view to detect antero-inferior instability impaction fractures at both the humeral and glenoid sides. Accurate quantification of bony abnormalities and detection of lesions to the soft-tissue stabilizers of the shoulder however require advanced cross-sectional imaging techniques.

## Introduction

The concept of joint instability refers to the loss of contact, partial or complete, transitory or permanent, between cartilaginous surfaces of a joint [1]. Shoulder instability can be classified according to the direction of instability, the type of clinical presentation, the etiology, mechanisms of injury, as well as anatomical lesions [2].

Imaging of the presumably unstable shoulder aims at detecting anatomical lesions related to the instability, locating them (hereby defining the direction of instability), and quantifying them. These parameters will also serve as prognostic factors and help to guide the management [1,2,3,4]. Conventional radiography is the first step of the diagnostic workup, since it allows the detection of osseous abnormalities following dislocations. However, advanced imaging techniques will be required to accurately quantify these osseous abnormalities, to detect associated lesions of soft tissue stabilizers of the shoulder (chondrolabral and capsuloligamentous lesions). Of note, congenital osseous abnormalities predisposing to shoulder instability (such as glenoid dysplasia) are relatively rare, although their incidence might be higher than previously thought [5,6].

The goal of this short review paper is to: discuss the optimization of radiographic views to detect osseous abnormalities in shoulder dislocation, present radiographic signs of previous dislocation, and review a few pitfalls on radiography.

## Antero-inferior shoulder instability

### Definition

Antero-inferior instability of the shoulder refers to a group of various entities that associate a translation of the humeral head relative to the glenoid, both in the anterior and the inferior directions. The relative importance of these anterior and inferior components depends on multiple parameters such as the direction of external constraints causing the dislocation, the position of the arm during the macro- or micro-traumatisms, and the status of the rotator cuff tendons or muscle tone.

Osseous abnormalities may occur at the sites of contact between the humerus and the glenoid. Although more severe fractures such as displaced comminutive fractures are possible, in the most typical case, dislocation may lead to an impaction fracture at the contact zones of the postero-superior humeral head (called the Hill-Sachs lesion) and the antero-inferior glenoid rim (called the Bankart lesion) [7,8,9].

### Bone Abnormalities On The Humeral Side: Hill-Sachs Lesions

**Figure 1:**
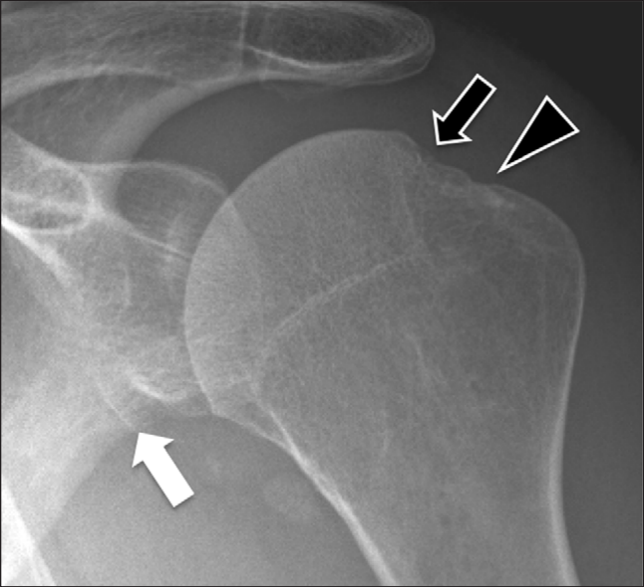
Hill Sachs lesion (black arrow) seen as a cortical depression of the postero-superior aspect of the humeral head. This depression is located medial to the head-neck junction (arrowhead). A Bankart lesion (fracture of the antero-inferior aspect of the glenoid rim) is also seen (white arrow).

**Figure 2:**
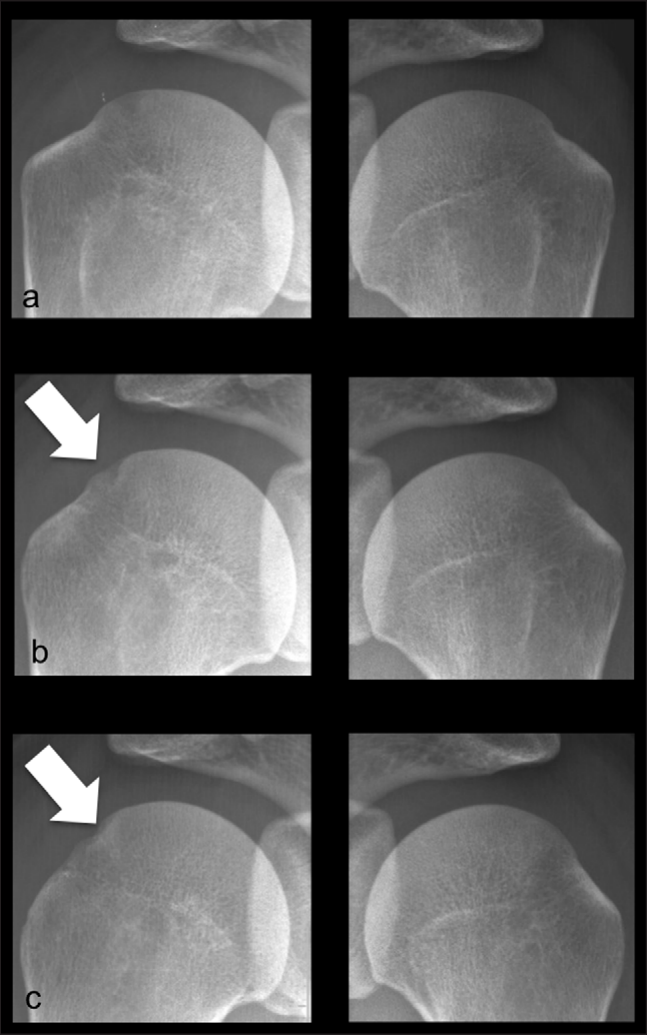
Bilateral frontal shoulder radiographs obtained with varying degrees of rotation of the humeral head, from neutral **(a)** to internal rotation **(c)**, without any modification to the beam angulation. The Hill-Sachs lesion (arrow), is not visible in neutral rotation. The pathological nature of the cortical irregularity of the postero-superior aspect of the humeral head can be affirmed in view of the normal aspect of contralateral asymptomatic left side.

The location of Hill-Sachs lesions varies depending on the position of the arm and the direction of the forces applied during trauma. In case of inferior displacement of the humerus, the position of Hill-Sachs lesions will be relatively cranial, whereas in more anterior displacement, it will be more posteriorly located. Most typically however, the lesion is located at the postero-superior aspect of the humeral head (Figure 1, 2) (Table 1).

**Table 1:** Impaction fractures on humeral and glenoid sides related to antero-inferior shoulder dislocation.

	Hill-Sachs Lesion	Bony Bankart lesion

**Bone**	Humerus	Glenoid
**Location**	Postero-superior	Antero-inferior
**Detection at radiography**	Relatively easy	Difficult
**Radiographic view**	Garth view	Garth view
**Diagnostic value**	+++	+++
**Prognostic value**	+	+++
**Value of CT/MRI**	+ (Quantification of bone loss, “engaging” or “off-track” lesions [21,27]	+++ (Quantification of bone loss)

**Figure 3:**
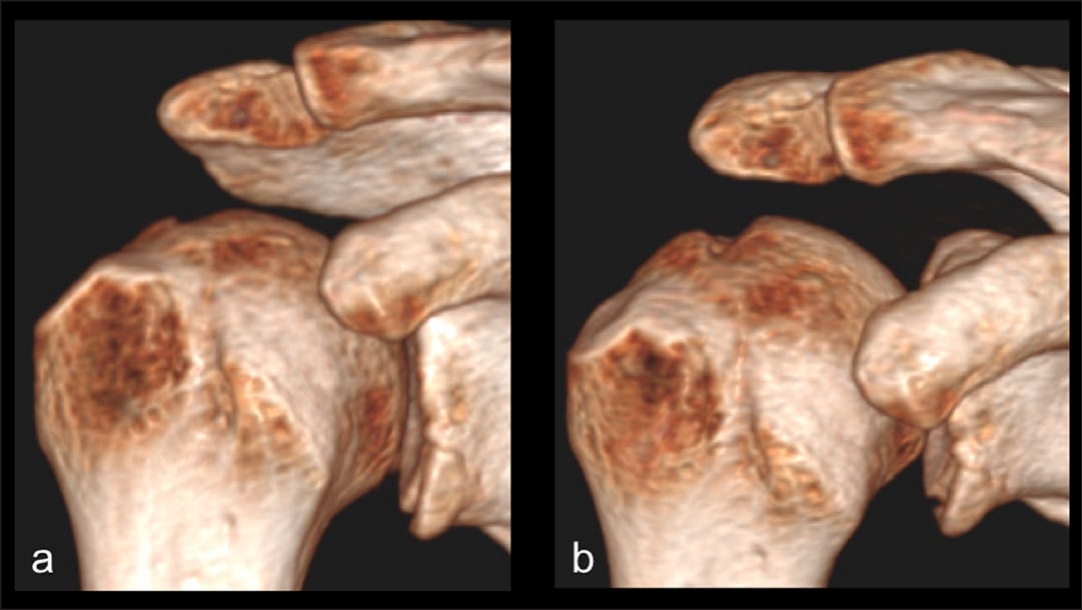
Varying visibility of the of the Hill-Sachs lesion depending on the angulation of the X-ray beam. Surface rendering reformats of the shoulder simulating varying degrees of angulation of the X-ray beam (as seen by the varying aspect of the acromion). With an ascending beam **(a)**, the Hill-Sachs lesion is almost not visible. With a descending beam **(b)**, the lesion is obvious.

**Figure 4:**
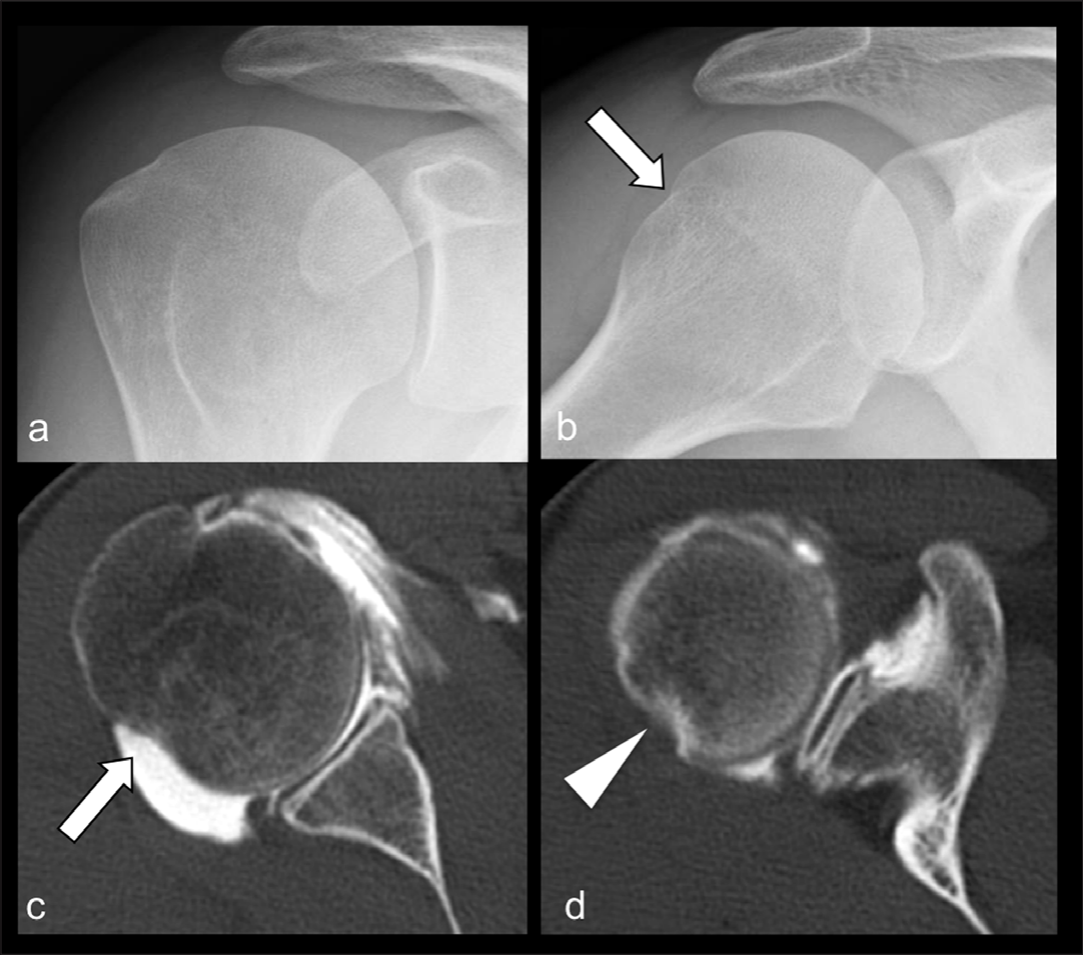
Frontal view of the shoulder in neutral rotation **(a)** and abduction/internal rotation **(b)** The depression (arrow) on the posterior head-neck junction of the humerus is a normal finding. The CT arthrogram axial image going through the mid third of the glenoid **(c)** shows the cortical depression with a large diameter base (arrow). The adjacent trabecular bone has a normal appearance. A CT arthrogram axial image through a more cranial part of the humeral head and the coracoid process **(d)** shows a depression with a smaller diameter base (arrowhead), corresponding to a Hill-Sachs lesion with sclerosis of the adjacent trabecular bone.

Due to its location, the radiological view that is most sensitive for the detection of the lesion is a triple oblique frontal view of the shoulder, as described by Garth et al. [10]. The patient has his/her shoulder internally rotated along the side, the beam is oriented externally and caudally at 45° (Figures 3 and 4). If necessary, the patient can lean forward. The Hill-Sachs lesion is less often seen on the frontal view of the shoulder in external rotation, on which the X-Ray beam is tangent to antero-superior aspect of the humeral head, often spared. In fact, the visibility of the Hill-Sachs lesion on this frontal view in external rotation is a sign of a greater depth of the fracture and is used as a negative prognostic factor (i.e. in the Instability Severity Index Score) [11].

**Figure 5:**
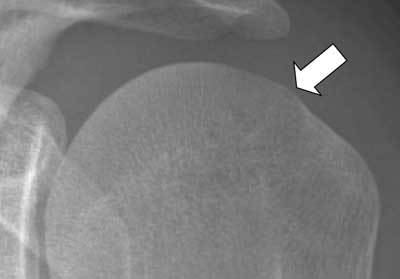
Variant of Hill-Sachs lesion in the form of a bony protuberance (arrow) rather than a depression, corresponding to its elevated margin, to which the beam is tangent.

**Figure 6:**
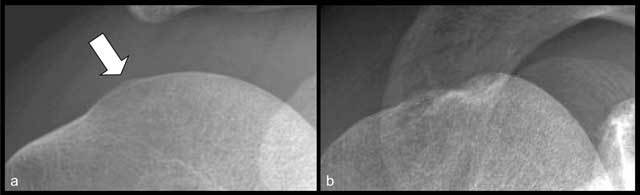
Variant of Hill-Sachs lesion on the frontal view in neutral rotation **(a)**, in the form of an “osteophyte-like” protuberance, in relation to the large depressed Hill-Sachs lesion visible with a descending beam **(b)**.

The Hill-Sachs lesion may have various appearances, either deep and angular, or shallow with smooth borders. Paradoxically, a Hill-Sachs lesion may sometimes appear as an “osteophyte-like” protuberance rather than a depression on radiographs, probably due to the fact that the X-Ray beam is, in those cases, tangent to the “elevated” margin, adjacent to the depressed area (Figures 5 and 6).

### Differential Diagnosis Of Hill-Sachs Lesions

**Figure 7:**
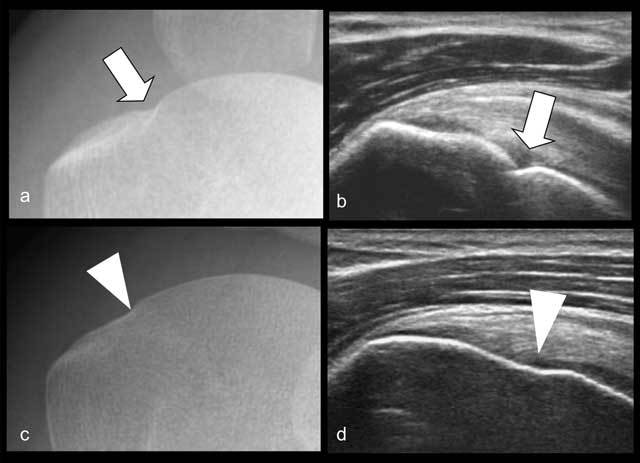
Interindividual variability of normal bony contours at the head-neck junction of the humeral head at radiography (**a** and **c**) and ultrasound (**b** and **d**). Marked depression (arrows in **a, b**) vs. smooth shallow depression (arrowheads in **c, d**).

Any deformity of the bone contours at the postero-superior aspect of the humeral head should not be considered a Hill-Sachs lesion. Actually, there are important interindividual variations in the normal osseous contours at the head-neck junction (Figure 7), but the aspect is usually symmetrical to the asymptomatic side (on similar radiographic projections). The physiological depression at the posterior aspect of the head-neck junction should not be confused with a Hill-Sachs lesion on abduction-internal rotation views of the shoulder (Figure 4) [12] This physiological depression is usually located caudal to the first 20mm of the humeral head, where Hill-Sachs lesions may occur.

Synovial inclusion cysts are extremely frequent, especially at the bare area of the posterior aspect of the humeral head [13,14]. They most likely result from hyperpression/friction between the bone and its capsulo-synovial environment, or from passage of synovial fluid into the bone. Synovial cysts are distinct from Hill-Sachs lesions by their location on the anatomical neck rather than on the humeral head and by their more lateral topography. They appear on radiographs as cortical (superficial) or trabecular (deep) defects, bordered by a thin sclerotic margin. Their shape is also usually distinct, rounder than Hill-Sachs lesions.

Marginal erosions associated with inflammatory erosive arthropathies are also located on the anatomical neck, just as synovial inclusions cysts [13].

Osseous abnormalities associated with the common mechanical enthesopathies can also lead to irregularities, or even focal deformities of the humeral head, but are located on the greater tubercle [15].

Finally, it is to be kept in mind that certain fractures of the greater tubercule can also be associated with shoulder dislocation, following in some cases the impact of the glenoid on the metaphyseal-epiphyseal junction of the humerus [16].

### Bone Abnormalities On The Glenoid Side: Bankart Lesions

Anteroinferior shoulder dislocation may lead to injury of the stabilizers of the shoulder on the glenoid side. These include injuries to the anterior or anteroinferior glenoid rim (called bony Bankart lesions), or to the labro-capsulo-ligamentous soft tissue stabilizers of the shoulder (called soft tissue Bankart lesions) [7]. Bony Bankart lesions are the only ones visible on conventional radiography. They will show as blunting, or as a fracture of the glenoid margin with an isolated bony fragment (Figure 1).

**Figure 8:**
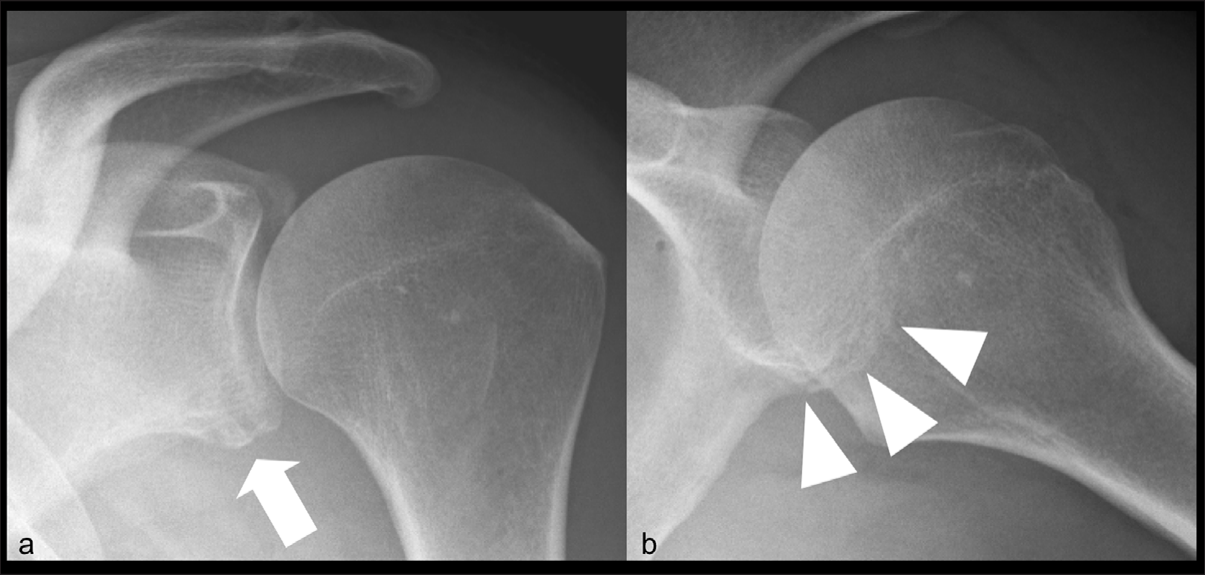
Bony Bankart lesion. Frontal view of the shoulder **(a)** showing irregularity of inferior glenoid rim (arrow) and loss of visualization of subchondral bone at most inferior aspect of glenoid. Frontal view in abduction, internal rotation of the shoulder **(b)** showing double contour of inferior margin of glenoid (arrowheads).

The detection of bony Bankart lesions on radiographs is not easy because they project on the glenoid in most cases (Figure 8). The strict AP view of the shoulder sometimes allows the detection of subchondral bone substance loss, because the X-ray beam is not strictly tangent to the glenoid, hereby allowing the differentiation between its anterior and posterior margins.

**Figure 9:**
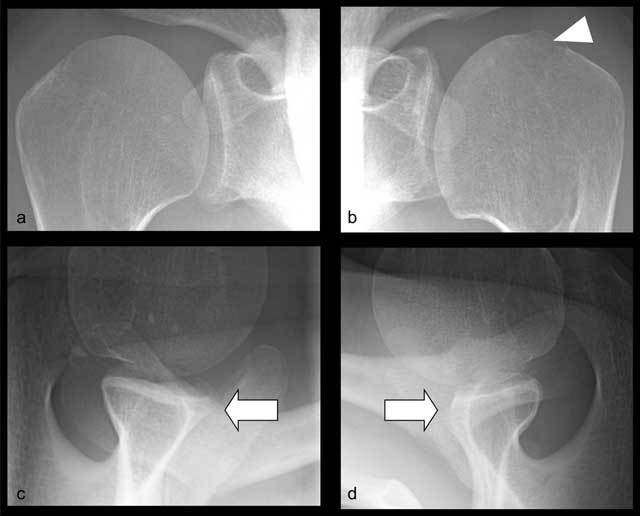
Bilateral frontal views of the shoulder showing protruberance at postero-superior aspect of humeral head on left side (arrowhead) that may correspond to Hill-Sachs lesion. Bilateral Bernageau views (**c, d**) showing normal glenoid margin on right side (arrow in c) and osseous substance loss with blunting of the antero-inferior margin of glenoid on left side (arrow in d), corresponding to a Bankart lesion.

**Figure 10:**
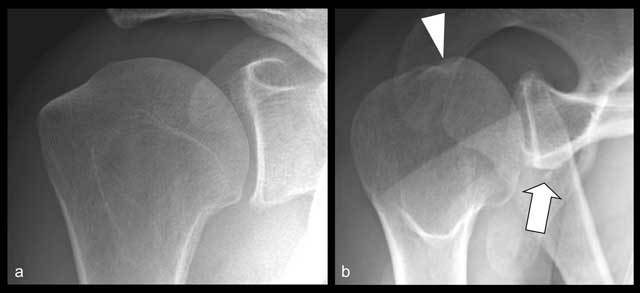
Hill-Sachs and Bankart fractures on Garth view. **(a)** Frontal shoulder radiograph (a) showing no abnormality. Garth view **(b)** showing a postero-superior depression of humeral head (arrowhead) and fracture of antero-inferior margin of glenoid (arrow).

The lateral view of the glenoid described by Bernageau is certainly the best view to detect Bankart lesions since the X-ray beam is tangent at the antero-inferior aspect of the glenoid (Figure 9) [17]. Both shoulders should be compared to analyze differences in the glenoid rim. Unfortunately, following a trauma, this view cannot be performed due to the difficulties of the patient in positioning its shoulder in abduction and external rotation. The Garth view is an excellent radiographic view for the visualization of Bankart lesions, and can be performed in the acute setting, since it can be obtained with the patient sitting or standing, without significant mobilization of the traumatized shoulder (Figure 10) [10]. On this view, the beam is tangent to both the postero-superior aspect of the humeral head and the antero-inferior glenoid rim, making it valuable for the detection of both Hill-Sachs and Bankart lesions.

Accurate quantification of the bone loss is however best performed with CT or MRI because these cross-sectional techniques have a better potential than radiography to quantify the bony substance loss [1,18,19,20,21,22,23,24,25]. Quantification of the glenoid bone loss may serve as a prognostic factor (higher risk of recurrence when greater bone loss), and to guide the choice between therapeutic options (capsulo-labral repair vs. bony stabilization through Bristow-Latarjet procedure) [11,26,27]. CT remains the gold-standard for the quantification of glenoid and humeral head fractures, despite the evolution of concepts, especially for bipolar bone loss (“engaging” or “off-track” Hill-Sachs lesions) [21,27,28].

### Differential Diagnosis Of Bankart Lesions

Isolated fractures of the glenoid anterior margin without history of dislocation are much less common than Bankart lesions. Advanced imaging techniques can show the normality of capsulo-labral structures in case of isolated glenoid fracture without dislocation.

**Figure 11:**
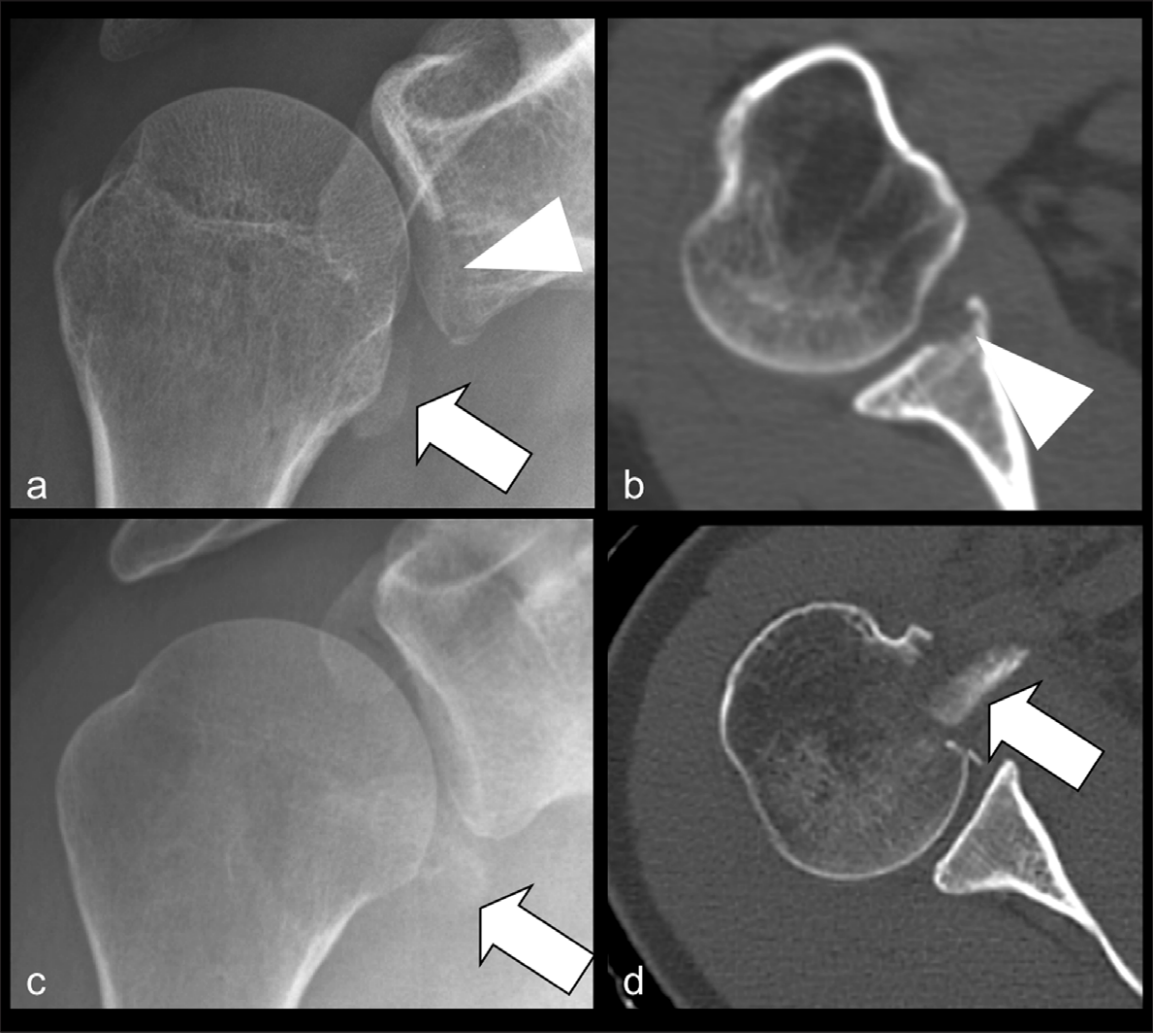
Radiographic **(a)** and CT **(b)** of bony Bankart lesion. Displaced bony fragment (arrow in a) and loss of subchondral bone plate (arrowheads). Radiographic **(c)** and CT **(d)** of fracture of lesser tubercle (arrows in c and d) mimicking a bony Bankart lesion at radiography (c).

The presence of an osseous fragment in the antero-inferior aspect of the glenoid may sometimes be due to an isolated fracture of the lesser tubercle of the humerus (Figure 11), not to be confused with a Bankart lesion. CT can correct the diagnosis.

Secondary osteo-chondral nodules differ by their rounder shape, their cortical margins, and their more medial location in the sub-coracoid recess.

## Posterior Shoulder Dislocation

**Figure 12:**
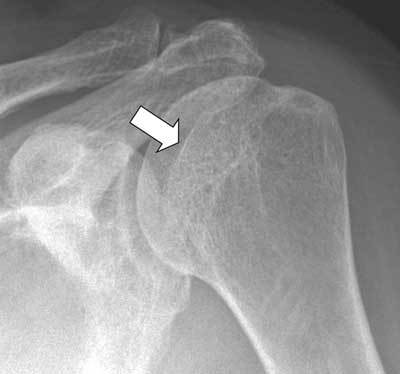
Frontal radiograph of shoulder showing double contour of humeral head (“trough sign”) (arrow) due to an reverse Hill-Sachs lesion secondary to history of posterior dislocation.

**Figure 13:**
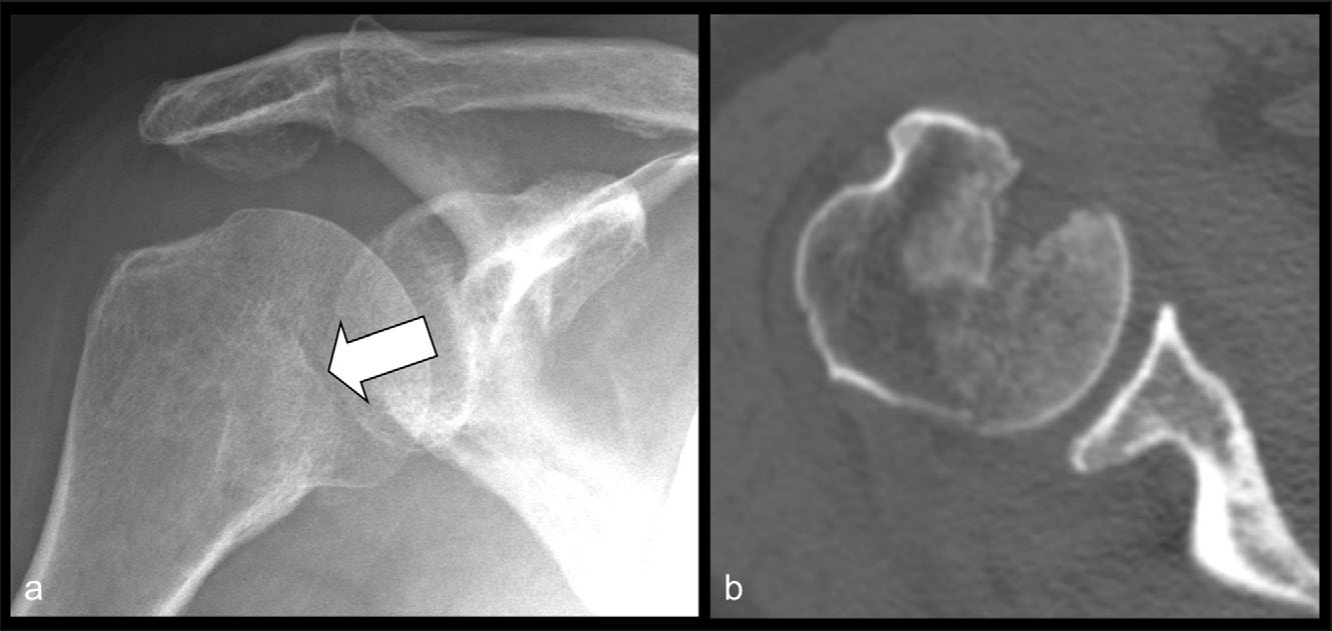
Patient with history of posterior shoulder dislocation. Radiograph **(a)** showing abnormal line on the humeral head (arrow). At CT, the deformity of the humeral head corresponds to the shape of the posterior margin of the glenoid.

In case of posterior shoulder instability, osseous abnormalities at the glenoid and humeral sides can be present, but their location varies. The impact fracture of the humerus lies anteriorly in case of posterior dislocation (and not at the postero-superior aspect of the humerus as after anterior dislocation), and is called a reverse Hill-Sachs lesion. Blunting or fracture of the glenoid lies at the posterior margin of the glenoid (and not antero-inferiorly), and is called a reverse Bankart lesion (Figure 12). The long axis of the reverse Hill-Sachs lesion, referred to as the “trough sign”, is often vertically oriented, due to the fact that posterior dislocation often occurs in the absence of abduction (acute spastic contraction of rotator cuff muscles in case of epileptic seizures or electrocution) (Figure 13). In general, the humeral head is displaced posteriorly and not postero-inferiorly (in case of anterior instability, anterior displacement of the humeral head is hindered by the presence of the coracoid process).

Reverse Hill-Sachs lesions are seen on the frontal view of the shoulder in neutral rotation and tends to fade with internal rotation of the shoulder. It can also be seen on the Bloom-Obata view [29]. The detection of these osseous lesions can be challenging and probably depends more on the acuity of the radiologist than on the obtained radiographic view. Due to the difficulties at diagnosing this lesion at radiography, advanced imaging techniques should be used in doubtful cases.

## Other Osseous Instablity Lesions

**Figure 14:**
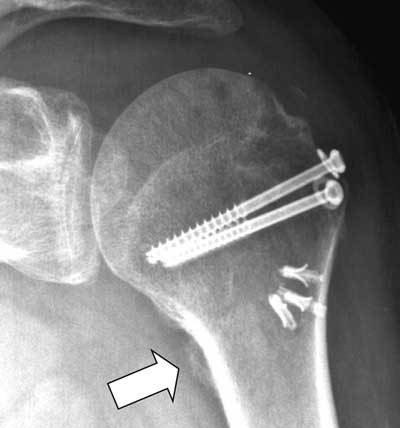
Radiograph showing ossification (arrow) at the insertion side of inferior glenohumeral ligament appeared after antero-inferior dislocation of the shoulder.

Glenohumeral ligaments participated to the stabilization of the shoulder and their insertions sites can be avulsed during dislocation. The avulsed cortical fragment or ossification close to the insertion sites may sometimes be seen (Figure 14) [30,31]. The exact rate of occurrence of such avulsion fracture is not known. They should not be confused with osseous fragments related to a Bankart lesion or to the avulsion of subscapularis tendon. This differentiation is sometimes difficult at radiography and advanced imaging may be needed. In some cases, ossifications at insertion sites may develop during the weeks after dislocation.

## Conclusion

Radiography remains pivotal to the workup of instability lesions of the shoulder, both in the acute as well as the chronic settings, with the goal of detecting osseous abnormalities and locating them in order to determine the direction of instability. In antero-inferior instability, Hill-Sachs lesions are often visible at radiography and should not be confused with various differential diagnoses, which are usually more laterally located. Bankart lesions are more difficult to detect at conventional radiography, but there are less false positives than for Hill-Sachs lesions. The Garth view represents an excellent radiographic view to detect antero-inferior instability impaction fractures at both the humeral and glenoid sides. Accurate quantification of bony abnormalities, the detection of lesions to the soft-tissue stabilizers and associated lesions (particularly of cartilage and the rotator cuff) however require advanced imaging techniques.
